# PC-3 prostate carcinoma cells release signal substances that influence the migratory activity of cells in the tumor's microenvironment

**DOI:** 10.1186/1478-811X-8-17

**Published:** 2010-07-13

**Authors:** Melanie J Voss, Bernd Niggemann, Kurt S Zänker, Frank Entschladen

**Affiliations:** 1 Institute of Immunology, ZBAF, Witten/Herdecke University, Stockumer Str. 10, 58448 Witten, Germany

## Abstract

**Background:**

Tumor cells interact with the cells of the microenvironment not only by cell-cell-contacts but also by the release of signal substances. These substances are known to induce tumor vascularization, especially under hypoxic conditions, but are also supposed to provoke other processes such as tumor innervation and inflammatory conditions. Inflammation is mediated by two organ systems, the neuroendocrine system and the immune system. Therefore, we investigated the influence of substances released by PC-3 human prostate carcinoma cells on SH-SY5Y neuroblastoma cells as well as neutrophil granulocytes and cytotoxic T lymphocytes, especially with regard to their migratory activity.

**Results:**

PC-3 cells express several cytokines and growth factors including vascular endothelial growth factors, fibroblast growth factors, interleukins and neurotrophic factors. SH-SY5Y cells are impaired in their migratory activity by PC-3 cell culture supernatant, but orientate chemotactically towards the source. Neutrophil granulocytes increase their locomotory activity only in response to cell culture supernantant of hypoxic but not of normoxic PC-3 cells. In contrast, cytotoxic T lymphocytes do not change their migratory activity in response to either culture supernatant, but increase their cytotoxicity, whereas supernatant of normoxic PC-3 cells leads to a stronger increase than that of hypoxic PC-3 cells.

**Conclusions:**

PC-3 cells release several signal substances that influence the behavior of the cells in the tumor's microenvironment, whereas no clear pattern towards proinflammatory or immunosuppressive conditions can be seen.

## Introduction

Today's understanding of the biology of tumors increasingly shows a crucial role of the microenvironment in the tumors' growth and the course of the cancer disease. This environmental interactions concern the development of new blood vessels in tumors (called neoangiogenesis), which was one of the first tumor-stroma interactions described in 1971 by Judah Folkman and colleagues [1], as well as similarly the development of new lymph vessels (lymphangiogenesis) [2], and the innervation of tumors, which we have termed neoneurogenesis [3,4]. Because of strong similarities to tissue growth and regeneration, tumors are regarded as wounds that do not heal [5]. A further argument for a comparison of tumors and wounds is the presence of cells of the immune system as well as fibroblasts infiltrating tumors being redolent of inflammatory conditions. Such parallels have already been observed by Virchow back in 1863, and this hypothesis of a non-healing wound has been seized and refined by Balkwill and Mantovani [6]. An inflammatory milieu is regarded as beneficial for a tumor in several aspects, concerning the growth as well as the migratory activity of tumor cells [7], which is a prerequisite for invasion and metastasis formation [8].

Two organ systems provide pro-inflammatory signal substances. Predominantly, there are of course the cells of the immune system, that release cytokines and chemokines which provoke inflammatory effect. However, the immune system is under the superordinate regulation of the nervous system, and the nervous system itself releases substances that play a role in inflammation. Thus, the above mentioned neoneurogenesis - the innervation of tumor tissue - might facilitate pro-inflammatory events in two ways: either directly by the release of neurotransmitters that act on the tumor cells (e.g. substance P, bradykinin, calcitonin gene-related peptide) [9], or indirectly by an action on leukocytes that release such factors in response.

But what causes the presence of leukocytes and the innervation of a tumor? It is well documented that tumor cells release a plethora of signal substances including chemoattractive molecules [10]. This release is a regulated process, e.g. by hypoxic conditions [11]. The lack of oxygen and nutrition provokes the tumor cells to release substances that initiate the above described three related processes: neoangiogenesis, lymphangiogenesis, and neoneurogenesis [3]. However, most studies on the tumor-environment interactions aim to the understanding of tumor vascularisation, and less attention has been paid to the mechanisms of tumor innervation and leukocyte infiltration. Therefore, we investigated by the use of the prostate carcinoma cell line PC-3 the tumor interactions with cells of the nervous system on the example of SH-SY5Y human neuroblastoma cells and with cells of the immune system on the example of neutrophil granulocytes and cytotoxic T lymphocytes (CTLs), especially with regard to the migratory activity of these cells. Migration is one essential cell function for nerve cells to innervate the tumor and for leukocytes to extravasate from the blood and infiltrate the tumor tissue.

## Methods

### Cell isolation and cell culture

Human CTLs and neutrophil granulocytes were isolated from peripheral blood of voluntary healthy donors as described previously [12]. Heparinized blood was diluted with PBS (1:1.7). Neutrophil granulocytes together with erythrocytes were separated from the lymphocyte-containing peripheral blood mononuclear cell fraction by a density gradient centrifugation on lymphocyte separation medium (LSM 1077; PAA, Pasching, Austria). Subsequently, the neutrophil granulocytes were further isolated from the pellet of the centrifugation. The pellet was mixed with platelet-depleted serum from the same blood donor and diluted by 1:1.3 with a high molecular weight dextran solution (Macrodex; Fresenius, Bad Homburg, Germany) containing 0.01 M EDTA. After 3 hours, the supernatant containing granulocytes was isolated and remaining erythrocytes were removed by a hypotonic lysis with 0.3% sodium chloride on ice. The neutrophil granulocytes were used for experiments immediately after isolation.

The CTLs were positively selected from the mononuclear cell fraction of the density gradient centrifugation by immunomagnetic beads, which were coated with mouse anti-human CD8 monoclonal antibodies (Dynabeads; Invitrogen, Karlsruhe, Germany). The mononuclear cell fraction was incubated with the beads for 10 minutes at 4°C. Bead-bound cells were isolated by eight times washing with Dulbecco's PBS (PAA) in a magnetic field. After that, the beads were removed from the isolated CTL using Detachabeads (Invitrogen) at incubation for 45 minutes at 20°C. The CTLs were kept in culture overnight with RPMI culture medium (PAA) containing 10% heat-inactivated fetal calf serum (PAA) and 1% penicillin/streptomycin solution (50 U/ml and 50 μg/ml, respectively; GIBCO, Eggenstein-Leopoldshafen, Germany) at 37°C in a humidified atmosphere containing 5% CO_2_.

The isolated CTLs were activated by anti-CD3 and anti-CD28 antibodies (both BD Pharmingen, Erembodegem, Belgium) as described previously [13]. Twenty-four-well plates were coated with 10 μg/ml of each of these antibodies in 1 ml PBS at 4°C over night. Cells were seeded at a concentration of 8 × 10^5 ^cells/ml in culture medium as described above. The cells were incubated for three days, with the daily addition of 150 ng/ml IL-2 (Invitrogen, Nivelles, Belgium). Maximum activation of the cells (as measured by the activation markers CD25 and CD45R0) was reached after four days, but the granularity (as measured by the sideward scatter) reached the maximum already after three days and decreased to day four.

PC-3 human prostate carcinoma cells (DSMZ, Braunschweig, Germany) were cultured in HAMs and RPMI medium (1:1) containing 10% heat-inactivated fetal calf serum (PAA) and 1% penicillin/streptomycin solution (50 U/ml and 50 μg/ml, respectively; GIBCO) at 37°C in a humidified atmosphere containing 5% CO_2_. Hypoxia was induced by a stepwise oxygen-deprivation in hypoxia-chambers (Billups-Rothenberg, Del Mar, CA): PC-3 cells were kept two days at 10% O_2_, one day at 5% O_2_, and one day at 1% O_2 _with constantly 5% CO_2 _and the remainder being N_2_.

SH-SY5Y human neuroblastoma cells (ATCC, Rockville, MA) were cultured in DMEM medium (PAA) containing 10% heat-inactivated fetal calf serum (PAA) and 1% penicillin/streptomycin solution (50 U/ml and 50 μg/ml, respectively; GIBCO) at 37°C in a humidified atmosphere containing 5% CO_2_. The cells were differentiated to fully neurotrophic factor-dependent neuron-like cells by treatment for five days with 10 μM retinoic acid (RA; Sigma-Aldrich, Taufkirchen, Germany) and used for experiments immediately after differentiation.

### Cell migration experiments

We performed our conventional three-dimensional, collagen-based migration assay as described in detail previously [12]. In brief, a suspension of 2.5 × 10^5 ^leukocytes or 8 × 10^4 ^SH-SY5Y cells or PC-3 cells in 50 μl RPMI was mixed with 100 μl of a buffered collagen solution (pH 7.4), containing 1.67 mg/ml bovine collagen type I (Invitrogen, Cohesion Technologies, Palo Alto, CA). The suspension was filled into self constructed migration chambers, which consisted of a microscopic glass slide, wax walls, and a cover slip on top. After polymerization of the collagen at 37°C in a humidified 5% CO_2 _atmosphere, concerning the leukocytes, the remaining chamber volume was filled with medium containing the investigated substances (medium conditioned with substances released by normoxic or hypoxic PC-3 cells), or, concerning the SH-SY5Y cells a second collagen matrix was applied next to the first one in the same manner as described above. This second collagen matrix contained 8 × 10^4 ^PC-3 cells. The migration of the cells was recorded by time-lapse videomicroscopy for 1 hour (leukocytes) or for 15 hours (SH-SY5Y and PC-3 cells) at 37°C. The paths of 30 randomly selected cells were digitized by computer-assisted cell tracking and the part of migratory active cells was calculated for each one minute or 15 minute interval. Statistically significant changes were calculated from the mean migratory activities in the steady state phase using the unpaired and undirected Student's *t *test; p-value lower than 0.05 was regarded as statistically significant.

### Gene expression profiling

In order to analyze changes of the gene expression in PC-3 cells in response to SH-SY5Y cells, these cells were co-cultured without having direct cell-cell contact. For these experiments cell culture inserts for 6-well plates were used (8.0 μm pore size; Becton Dickinson, Le Pont De Claix, France). PC-3 cells (5 × 10^5^) were cultured in the bottom of the 12-well plates, and SH-SY5Y cells (2 × 10^5^) were cultured in the inserts. Empty inserts served as control. After 24 hours incubation, the total RNA of the PC-3 cells was isolated using a NucleoSpin RNA II kit (Machery&Nagel, Dueren, Germany). Real-time analysis of the gene expression of PC-3 cells (1 μg RNA per sample) was performed using the RT^2 ^Profiler PCR Array System (SABiosciences, Frederick, MD) according to the manufacturer's protocol and an Abi Prism 7700 Sequence Detector (Applied Biosystems, Weiterstadt, Germany). Gene sets of the human growth factor kit and the human tumor metastasis kit were used.

### Cyotoxicity assay

The lytic activity of CTLs was analyzed by a colorimetric, non-radioactive cytotoxicity assay (Promega, Mannheim, Germany), which quantitatively measures the release of lactate dehydrogenase (LDH) after cell lysis according to Jurisic *et al*. [14], and as described previously [15]. K562 human myeloid leukemia cells (DSMZ, Braunschweig, Germany) were used as target cells. In the assay, the PC-3 cell culture supernatants were added to the CTLs immediately before incubation with target cells. Non-conditioned medium served as control. The effectors were used at an effector:target ratio of 10:1. Cells were co-incubated for 4 h at 37°C, the supernatants were collected and the LDH release was evaluated by a colorimetric reaction (absorbance at 490 nm). The percentage of lysis was calculated by comparing the LDH released for each lysis condition maximal LDH release.

## Results

### Effects of signal substances derived from PC-3 cells on SH-SY5Y neuroblastoma cells

**Figure 1:**
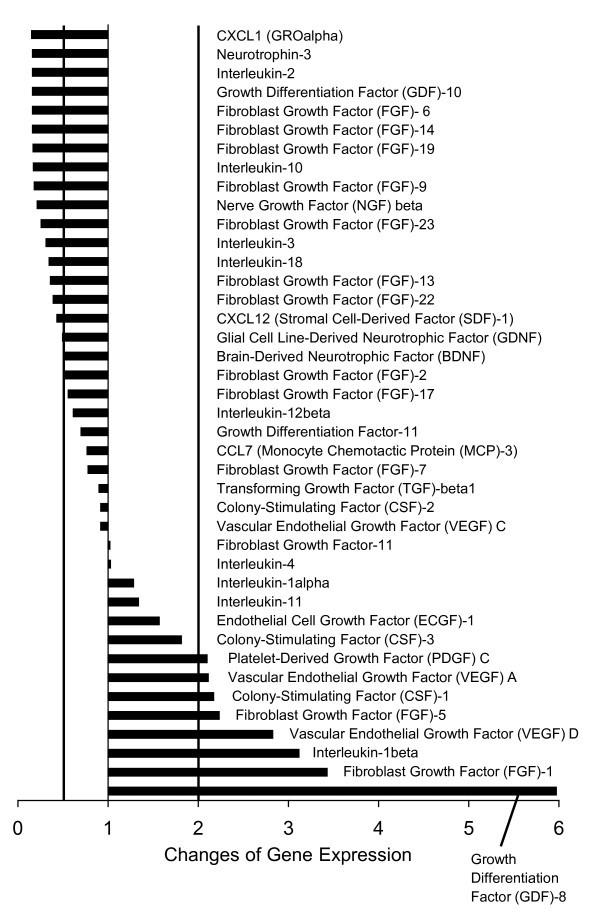
**Gene expression analysis of cytokines and chemokines in PC-3 human prostate carcinoma cells in response to SH-SY5Y cell culture supernatant**. Downregulation of a gene to less than the half of the expression in control cells, and upregulation to more than the double were accepted as significant changes (vertical lines).

**Figure 2:**
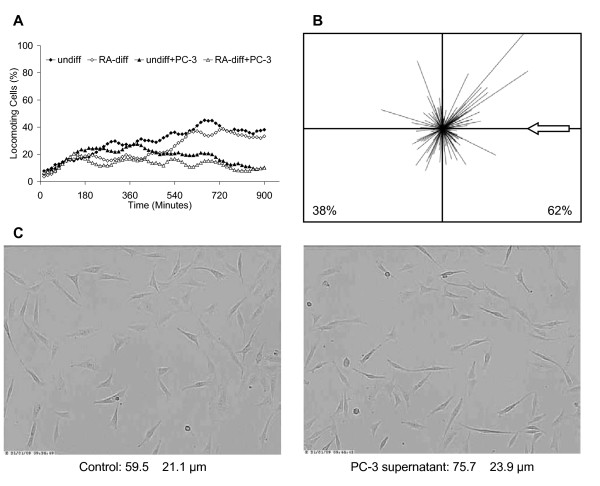
**Changes of the migratory activity of SH-SY5Y human neuroblastoma cells in response to PC-3 cell-conditioned medium**. A and B: the migratory activity and directionality of migration was analyzed by means of the collagen-based three-dimensional assay utilizing time-lapse videomicroscopy. In A, the graph shows the mean value of three independent experiments (90 cells were analyzed per sample); in B, the graph shows the summary and mean value of four independent experiments (120 cells were analyzed). The arrow indicates the source of substances released by PC-3 cells. C: the images show the morphology of the SH-SY5Y cells. Mean values and standard deviation of the cells' lengths were calculated from sizing 120 cells per sample.

We have reported previously that PC-3 prostate carcinoma cells have influence on the SH-SY5Y neuroblastoma cells with regard to their differentiation by the release of signal substances [16]. However, we have in this previous work focussed on the expression of some neuronal growth factors. Therefore, we analyzed in a much broader range by real-time RT-PCR, which signal substances are expressed by tumor cells. We performed these analyses either without any stimulus or with culture supernatant that was conditioned with SH-SY5Y neuroblastoma cells. PC-3 human prostate carcinoma cells express several cytokines of the growth factor and interleukin group as well as chemokines (Fig. 1). With our arrays, we have detected 41 of these substances, whose expression is differentially regulated upon treatment with SH-SY5Y cell culture supernatant. Most noticeable is the large number of fibroblast growth factors (FGFs) that are expressed. FGF-1, -5 and -11 are down-regulated after 24 treatment of the PC-3 cells with SH-SY5Y conditioned medium, whereas the expression of FGF-2, -7, -9, -13, -17, -22 and -23 remains unchanged, and the expression of FGF-9, -14 and -19 increases (Fig. 1). Furthermore, PC-3 cells express growth differentiation factors (GDF-8, -10, -11), vascular endothelial growth factors (VEGF A and C), as well as growth factor that are more specific for the nervous system, i.e. the nerve growth factor (NGF) beta, the brain-derived neurotrophic factor (BDNF), the glial cell line-derived neurotrophic factor (GDNF), and neurotrophin-3. Among the interleukins are IL-1alpha and beta, -2, -3, -4, -11, -12beta and -18. In summary, the PC-3 prostate carcinoma cells express a bunch of signal molecules that might influence the growth and differentiation of nerve cells and that furthermore facilitate the development of an inflammatory milieu in the tumor environment. Therefore, we investigated the migration of SH-SY5Y cells under the influence of the PC-3 cells' secreted factors. Surprisingly, the migratory activity of SH-SY5Y was strongly impaired under influence of PC-3 cell conditioned medium (Fig. 2A). The migratory activity of undifferentiated SH-SY5Y was significantly (p < 0.001) reduced from 37.6% ± 7.1% to 15.0% ± 6.4% migrating cells, and the migratory activity of SH-SY5Y cells that were differentiated with retinoic acid was significantly (p < 0.001) reduced from 39.0% ± 8.0% to 11.1% ± 4.2% migrating cells, as was analyzed from the migratory activity within the time-frame 720 to 900 minutes (Fig. 2A). However, although the overall migratory activity was reduced, the SH-SY5Y orientated chemotactically towards the PC-3 cells, when those cells were present in an adjacent collagen matrix (Fig. 2B). Furthermore, the spreading of SH-SY5Y cells increased in response to PC-3 culture supernatant (Fig. 2C). The cells were significantly (p < 0.001) longer by 27% when treated with the PC-3-conditioned medium. In summary, PC-3 cells mutually interact with SH-SY5Y cells: PC-3 cells release signal substances that affect the SH-SY5Y cell migration, and the SH-SY5Y in turn modify the expression of these signal substances in PC-3 cells.

**Figure 3:**
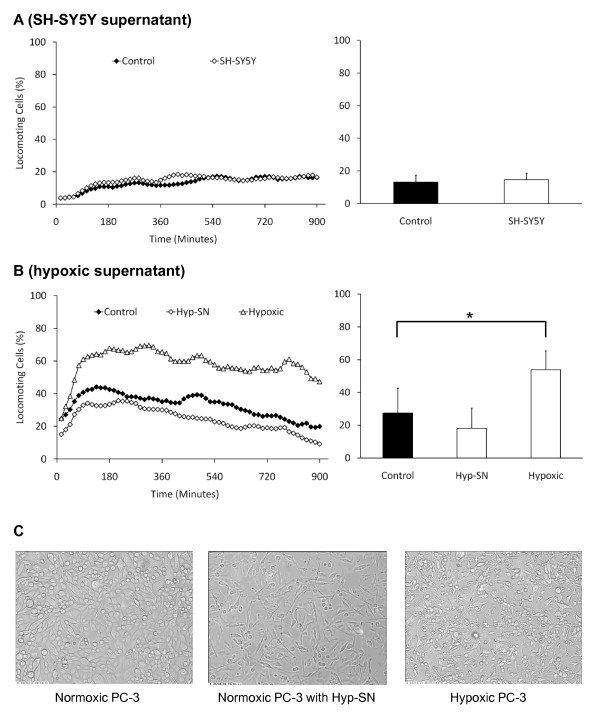
**Migratory activity of PC-3 prostate carcinoma cells in response to cell culture supernatant of SH-SY5Y cells (A), and hypoxic PC-3 cells (B)**. The left graphs show the time-course of the migratory activity. The right graphs show the corresponding time-average and the standard deviation of four independent experiments with cells from different donors (120 cells have been analyzed per sample). SN = supernatant (of cell cultures). Asterisks indicate statistically significant changes. In C, the images show the morphology of the cells corresponding to the conditions of (B)

The raising question is, whether the PC-3 cells themselves might be affected in their migratory activity by their own signal substances or by those of the SH-SY5Y cells. Cell culture supernatant of SH-SY5Y cells had influence on the gene expression, as shown in Fig. 1, but did not influence the migratory activity of the PC-3 cell (Fig. 3A). Furthermore, we have reported previously that hypoxic conditions lead to a change of the released substances' profile of PC-3 cells towards more inflammatory conditions similar to SH-SY5Y cells, e.g. by an increase of the release of interleukin-1 [17]. However, in this previous publication we have shown that oxygen-deprived PC-3 cells significantly increase their migratory activity [17]. We have extended these experiments on PC-3 cells, and have added samples of normoxic PC-3 cells that were treated with cell culture supernatant of hypoxic PC-3 cells in order to see if these released factors may work in an autocrine manner (Fig. 3B). Interestingly, culture supernatant of hypoxic PC-3 cells slightly reduced the migratory activity of PC-3 cells that were kept under normoxic conditions from 27.4% ± 15.4% to 18.2% ± 12.4% migrating cells, whereas hypoxic PC-3 cells significantly (p = 0.019) increase their migratory activity to 54.0% ± 11.5% migrating cells (Fig. 3B). This result is insofar interesting, as the released signal substances of hypoxic PC-3 cells are not the stimulus for the migratory activity. Thus, oxygen-deprivation must directly induce an intracellular signalling pathway that stimulates migration. However, the cell culture supernatant has an effect on the morphology of the PC-3 cells (Fig. 3C). Under normal culture conditions, PC-3 cells are spherical or spindle-shaped. When treated with cell culture supernantant of hypoxic PC-3 cells, the normoxic cells change their morphology and look similar to PC-3 cells that are kept under hypoxic conditions. The cells develop very long pseudopodia (Fig. 3C). The observed inhibition of PC-3 cell migration by hypoxic conditioned cell culture medium is not due to an over-stimulation by high concentrations of signal substances, since dilutions of the conditioned medium by 1:10 and 1:100 had no effect on the migratory activity (data not shown).

### Effects of signal substances from PC-3 cells on leukocytes

An important skill of cells that are involved in wound healing and inflammation is the ability to migrate. Leukocytes have to enter these wounded or inflamed sites from the blood stream by passing the vascular endothelial cells in order to eliminate pathogens and fully accomplish this tissue's functionality. As mentioned above, leukocytes are frequently found in tumors. Neutrophil granulocytes and macrophages can make up to 50 percent of the total tumor mass in breast carcinoma [18], and neutrophil granulocytes are supposed to play a role in inflammation-induced angiogenesis [19,20].

**Figure 4:**
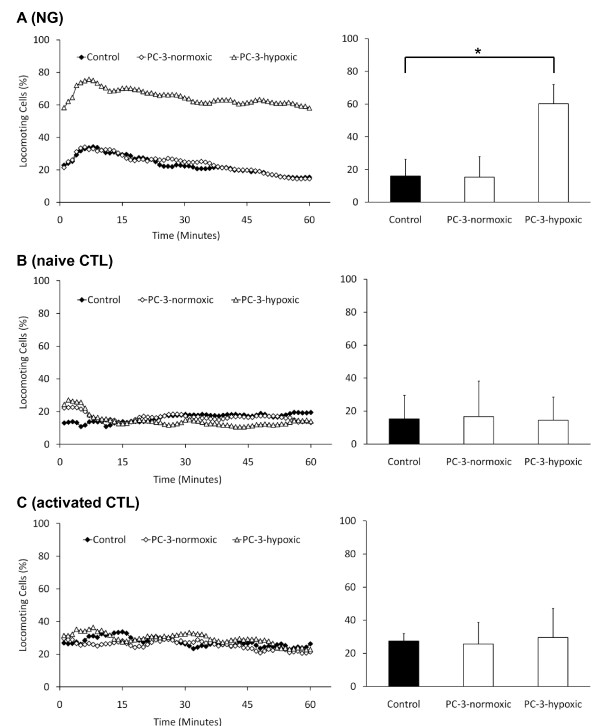
**Migratory activity of neutrophil granulocytes and CTLs in response to cell culture supernatant of either normoxic or hypoxic PC-3 cells**. The left graphs show the time-course of the migratory activity. The right graphs show the corresponding time-average and the standard deviation of three independent experiments with cells from different donors (90 cells have been analyzed per sample). Asterisks indicate statistically significant changes.

**Figure 5:**
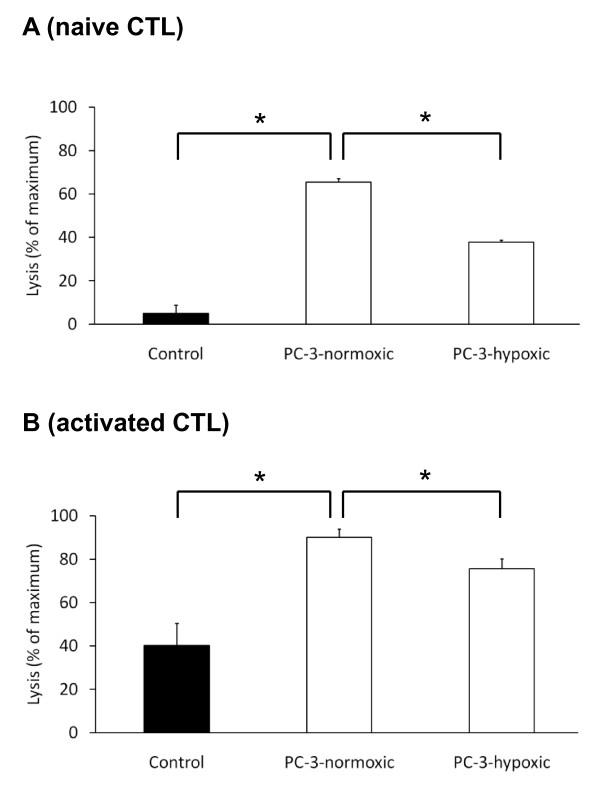
**Lysis of naïve (A) and activated (B) CTLs in response to PC-3 cell culture supernatants**. The cytotoxic activity of the CTLs was analyzed by the release of lactate dehydrogenase from lysed K562 cells. The columns show mean values and standard deviation of four (A) and three (B) experiments. Asterisks indicate statistically significant changes.

Cell culture supernatant of PC-3 cells that were kept under normal conditions had no effect on the migratory activity of neutrophil granulocytes (Fig. 4A), but when the PC-3 cells were kept under hypoxic conditions, the migratory activity of the neutrophil granulocytes was significantly (p < 0.001) increased from 16.0% ± 10.3% to 60.2% ± 11.8% migrating cells. In contrast, the migratory activity of neither naïve nor activated CTLs was influenced by normoxic or hypoxic cell culture supernatant, whereas the overall migratory activity of activated CTLs was higher than of naïve cells (27.4% ± 4.7% compared to 15.3% ± 14.3% migrating cells; Fig. 4B and 4C). However, the cytotoxic activity of these cells was significantly influenced by signal substances of PC-3 cells (Fig. 5A and 5B). Cell culture supernatant of PC-3 cells that were kept under normoxic conditions significantly (p < 0.001) increased the lytic activity of naïve CTLs from 4.9 ± 3.8 percent of the maximal lysis to 65.5 ± 1.6 percent. Hypoxic cell culture supernatant significantly (p < 0.001) reduced this activity down to 37.8 ± 1.0 percent (Fig. 5A). Activated CTLs showed an overall higher lytic activity than naïve CTLs. The lysis of these cells significantly (p = 0.001) increased from 40.3 ± 11.1 percent of the control to 90.1 ± 3.8 percent with normoxic PC-3 cell culture supernatnant. Hypoxic cell culture supernatant significantly (p = 0.013) reduced this activity down to 75.6 ± 4.5 percent (Fig. 5B). In summary, PC-3 cells release various signal substances under normoxic and hypoxic conditions, which have divergent effects on the function of leukocytes.

It would be of course of interest, if in a more complex setting the mutual interaction of SH-SY5Y cells with PC-3 cells might also affect the function of the leukocytes. However, such experiments were started but brought no definite result so far. As one can understand, this is largely based on the fact, that we can treat either cell type (SH-SY5Y cells or PC-3 cells) with culture medium of the other cell type, respectively, but we can subsequently not separate the substances released by these two cell types. Therefore, we have always a mixed effect on the leukocytes with high variations and have thus abandoned from presenting results on this.

## Discussion

Although it is currently a focus in cancer research, how hypoxia changes the profile of expressed and released signal substances, especially with regard to neoangiogenesis [21-23], it is frequently reported that tumor cells release several signal substances even under normoxic conditions either constitutively or in response to other stimuli than hypoxia [24-27] including substances that have especially impact on nerve cells [28]. We show herein that the PC-3 cells change the expression profile of signal substances not only in response to hypoxia, as reported previously [17], but also in response to other cells such as the nerve-like SH-SY5Y cells. Such a mutual exchange of signals might facilitate the innervation of the tumor. Our gene expression analysis has shown that several FGFs are expressed, which are known to be involved at least in neoangiogenesis and lymphangiogenesis [29,30]. As documented before, these two processes have a common regulation with neoneurogenesis [3], and we thus argue that the nerve cells themselves contribute to the regulation of tumor innervation. It has been shown before that tumors release neurotrophic factors [31-33], and the presence of neuroendocrine markers in tumors is correlated with a poor prognosis in colorectal cancer [34,35]. It is noteworthy that the neurotransmitter norepinephrine strongly enhances the migratory activity of PC-3 cells [36], and leads to an increased metastasis development in mice [37]. Furthermore, prostate cancer cells invade and spread along nerve fibres, a process known as perineural invasion, which is correlated with a worse outcome in patients [38,39]. Therefore, the innervation of a tumor might support tumor progression by the release of promigratory neurotransmitters such as norepinephrine and the guidance of cells away from the primary tumor. However, one final note is that the SH-SY5Y not constitutively release substances that induce migration of PC-3 cells, as shown in Fig. 5A.

As written above, tumors are often described as wounds that do not heal [8]. In contrast, tumors may induce immune dysfunction [40], e.g. by the release of immunosuppressive substances such as interleukin-10 [41], or by an active counter-attack that induces apoptosis in the tumor-infiltrating leukocytes [42]. In addition to the above discussed induction of metastasis formation, the innervation of tumors might support a proinflammatory milieu by the release of the according neurotransmitters. For example, peptidergic nerve fibres have been found in esophageal and cardiac carcinoma [43]. In that study, substantial amounts of substance P have been found, which is a key mediator of inflammatory processes [44].

With regard to the interaction of the PC-3 cells with leukocytes, the substances released by the tumor cells do not provide a clear picture that points either to an inflammation-like immune stimulation or a to tumor-promoting immunosuppression in terms of a tumor escape mechanism. The expression of interleukin-10 is reduced in the presence of SH-SY5Y cells, whereas the expression of interleukin-1β is increased (Fig. 1). As reported previously, the expression of both interleukin-1α and β was enhanced under hypoxic conditions, too [17].

In terms of the leukocyte function, the migration of neutrophil granulocytes, but not of CTLs was stimulated solely by hypoxic PC-3 cell culture supernatant, whereas the normoxic cell culture supernatant has no effect on either cell type. This would be a sign of a rather inflammatory milieu, but in contrast, such a hypoxic milieu reduces the cytotoxicity of CTLs, which is simulated by the normoxic cell culture supernatant. However, a large number of substances that are released by the PC-3 cells have been described to modulate the function of immune cells, either alone or in combination. We are thus not able to attribute the observed effects to one single substance or a close circle of these substances. For example, the increase of interleukin-1β release in response to SH-SY5Y cells or hypoxia might explain the increase in the migratory activity of neutrophil granulocytes [45], but even other ligands whose expression has not been investigated here, might be responsible for this effect.

Finally, concerning the tumor cells themselves, there are some lines of evidence that the cells stimulate their migratory activity in an autocrine manner [46-49], including interleukin-1α and β [50], as well as FGF-5 [51], which are released by the PC-3 cells, too. Although the migratory activity of PC-3 cells significantly increases under hypoxic conditions, this is not due to such an autocrine effect (Fig.3B). In contrast, the substances released by hypoxic PC-3 cells slightly reduce the migratory activity of normoxic cells. Thus, hypoxia or its mediator, the transcription factor HIF (hypoxia inducible factor), directly activate an intracellular signalling pathway in the PC-3 cells, which induces migratory activity independent of an autocrine action of the released signal substances. In a broader view, this increase of migratory is important with regard to anti-angiogenic or angiostatic cancer treatment. Such treatments have been implemented in therapy protocols in order to cut off the tumor from nutrients and thereby stop its growth or ultimately provoke cell death. However, there is an increasing number of publications that report about an aggravation of the course of the cancer disease with respect to metastasis formation by the use of such therapeutics, and in light of these reports anti-angiogenesis has maybe to be revisited [52]. The herein presented stimulation of migratory activity by oxygen deprivation might deliver an explanation for these evil side-effects of anti-angiogenic drugs.

## Conclusions

It is well established that a growing tumor interacts with its microenvironment in order to accomplish nutrient supply, disposal of metabolites, and innervation. In this regard, a tumor behaves like any physiologically growing tissue. However, it still remains obscure, how these substances that are released by tumor cells in concert trigger an immune response. Hypotheses generally point to an inflammatory situation in tumors. Our results show that the signal substances released by PC-3 human prostate carcinoma cells have the potential to stimulate the innervation of a tumor, and that various leukocyte functions are affected. However, we have not seen a clear picture towards a proinflammatory process. Our functional investigations rather provide a chaotic picture with regard to the regulation of the immune system, which supports a view of a tumor escape by immune dysregulation.

## Competing interests

The authors declare that they have no competing interests.

## Authors' contributions

MJV performed the experiments, BN supported the analysis of the cell migration data, MJV, KSZ and FE designed the study and analyzed the results, MJV and FE wrote the manuscript and made the figures. All authors have read and approved the final manuscript.
